# *APOE* genotypes are associated with the level of naturally occurring antibodies to amyloid-*β* in patients with Alzheimer’s disease

**DOI:** 10.3389/fnagi.2026.1673361

**Published:** 2026-02-10

**Authors:** Janardan P. Pandey, Aryan M. Namboodiri, Franca Rosa Guerini, Elisabetta Bolognesi, Milena Zanzottera, Roberta Mancuso, Simone Agostini

**Affiliations:** 1 Department of Pharmacology and Immunology, Medical University of South Carolina, Charleston, SC, United States; 2 IRCCS Fondazione Don Carlo Gnocchi, Milan, Italy

**Keywords:** Aβ, Alzheimer’s disease, *APOE*, humoral immune response, humoral immunity

## Abstract

Apolipoprotein E ε4 (*APOEε*4) allele is the strongest known genetic risk factor for Alzheimer’s disease (AD). Mechanisms underlying this association are incompletely understood. We aimed to determine whether *APOE* genotypes influenced the level of naturally occurring antibodies to amyloid-*β* (Aβ), a hallmark of AD, and whether anti-Aβ antibodies contributed to neurodegeneration, as measured by mini-mental state examination (MMSE) score. The study population consisted of 93 Italian AD patients. Results showed that *APOEε*4-carriers had significantly higher levels of anti-Aβ antibodies than non-carriers (*p* = 0.018). After adjusting for age, sex, Aβ levels in serum, and the MMSE scores, regression analyses showed marginal association between *APOEε*4 carrier status and the levels of anti-Aβ antibodies (*p* = 0.050). This is the first report of its kind and needs to be confirmed in a large multiethnic population.

## Introduction

The apolipoprotein E (*APOE*) gene, located on chromosome 19q33, is characterized by the segregation of three alleles—*ε*2, *ε*3, and *ε*4. *APOEε*4 increases the risk of developing late-onset Alzheimer’s disease (AD), while *APOEε*2 is protective, compared to the most common *APOEe*3 allele. One mechanism through which *APOEε*4 increases the risk of AD is by promoting aggregation and deposition of amyloid-*β* (Aβ), a hallmark of AD (Jackson et al., 2024). AD patients generate anti-Aβ autoantibodies, which have been shown to contribute to the reduction of Aβ plaque burden (Kellner et al., 2009). There are significant interindividual differences in the level of naturally occurring anti-Aβ autoantibodies, but no host genetic factors that might contribute to these differences have been identified. Identification and understanding of the host factors that influence naturally occurring immune responses to Aβ is an important prerequisite to successfully designing Aβ-based immunotherapeutic approaches against AD. Although monoclonal anti-Aβ antibodies approved for AD therapy have shown evidence of slowed clinical decline, they are associated with serious side effects, underscoring the need for a better understanding of the immunobiology of Aβ, which could lead to more efficacious alternative therapies (Jucker and Walker, 2023).

The aim of the present investigation was to determine whether the *APOE* genotypes influenced the level of naturally occurring antibodies to Aβ in AD patients, and whether anti-Aβ antibodies contributed to neurodegeneration, as measured by mini-mental state examination (MMSE) score.

## Materials and methods

### Patients

In total, DNA and sera were available from 93 AD patients, recruited by the Rehabilitative Neurology Unit of the IRCCS Fondazione Don Carlo Gnocchi, Milan, Italy. Patients were diagnosed as probable AD according to the NINCDS-ADRDA criteria (Mckhann et al., 2011). Patients were excluded if they suffered from malnutrition or vitamin deficiency syndromes, and recent introduction or dose modification of the following pharmacological treatments: cholinesterase inhibitor, memantine, antidepressant or antipsychotic drugs. Demographic and clinical data of the enrolled patients are presented in Table 1.

**Table 1:** Demographic, clinical, and laboratory data of the enrolled subjects.

Parameters		*APOEε*4-non-carriers	*APOEε*4-carriers	*p*-value
*N*		48	45	---
Gender (M: F)		16:32	19:26	*p* = 0.782
Age (years, mean ± SD)		77.28 ± 6.78	75.87 ± 5.68	*p* = 0.307
MMSE (adj, median and IQR)		20; 19.00–21.35	19; 18.33–22.12	*p* = 0.996
Educational level (years, mean ± SD)		8.05 ± 2.75	8.31 ± 3.93	*p* = 0.882
*APOE* genotype *N* (%)	2/2	1 (2.1)	0 (0.0)	---
	2/3	4 (8.3)	0 (0.0)	
	2/4	0 (0.0)	1 (2.2)	
	3/3	43 (89.6)	0 (0.0)	
	3/4	0 (0.0)	38 (84.4)	
	4/4	0 (0.0)	6 (13.3)	
Serum Aβ_1-42_ oligomer (pg/mL, median and IQR)		0.01 (0.01–0.01)	0.01 (0.01–1.55)	*p* = 0.083
Serum anti-Aβ antibodies (AU/μL, median and IQR)		10.52 (6.52–15.57)	14.25 (9.82–19.00)	*p* = 0.018

Aβ, amyloid-beta; *APOE*, Apolipoprotein E; IQR, Interquartile range; M, male; F, female; MMSE, mini mental state evaluation, adjusted; SD, standard deviation.

### *APOE* genotyping

*APOE* genotyping for the three major isoforms (*ε*2, *ε*3, and *ε*4) was done by RT-PCR, using pre-designed TaqMan™ probes (Thermo Fisher Scientific, Waltham, MA, USA)—C_904973 (rs7412) and C_3084793 (rs429358).

### Measurement of Aβ_1-42_

Aβ_1-42_ was measured in serum of all the enrolled subjects by using a commercial enzyme-linked immunosorbent assay (ELISA), according to the manufacturer’s instructions (cat. JP27719, IBL International, Hamburg, German). Briefly, 100 μL of serum samples diluted (1:50) with sample diluent were transferred into the pre-coated microwells and the plates were incubated overnight at 4 °C. After washing steps with washing buffer, 100 μL of labeled antibody were added to each well and incubated for 60 min at 4 °C. After re-washing step, 100 μL of chromogen solution were added to each well and incubated at room temperature for 30 min. Finally, 100 μL of stop solution were added to each well and the reaction stopped. The wells were read on a plate reader (Sunrise, Tecan, Mannedorf, Switzerland) and optical densities (OD) of wells were determined at 450 nm. Aβ_1-42_ concentration was expressed as pg/mL (sensitivity: 0.29 pg/mL).

### Measurement of anti-Aβ antibodies

Antibodies to Aβ_1-42_ oligomer were also measured by ELISA, using sera from AD patients. Aβ antigen was purchased from StressMarq Biosciences (Victoria, British Columbia). Ninety-six well microtiter plates were coated with Aβ antigen, 1 μg/mL in 100 μL phosphate-buffered saline (PBS), pH 7.4 overnight at 4 °C. Plates were washed with PBS containing 0.05% tween 20 (PBS-T) and blocked with PBS-T containing 5% bovine serum albumin (BSA) for 1 h at 37 °C. Plates were then washed and incubated with suitably diluted patient plasma (1:200) in duplicate wells. Plates were further washed and incubated with antihuman IgG HRP conjugate for 30 min. Finally, plates were washed and incubated with HRP substrate hydrogen peroxide along with 3,3′,5,5′-Tetramethylbenzidine as chromogenic substrate in citrate phosphate buffer (pH 5.5). Reaction was stopped after 20 min by the addition of 100 μL of 2 N HCl and the absorbance values at 450 nm were monitored in a BioTek ELISA reader. Wells containing plasma diluent buffer alone were used as blank. Absorbance values of blank wells were subtracted from the sample wells. Antibodies to Aβ were expressed as arbitrary units per microliter (AU/μL), after multiplying with the dilution factor.

### Statistical analyses

Statistical analyses were performed using the commercial MedCalc Statistical Software package (Version 11.5.0.0; Ostend, Belgium) and R. Due to relatively small sample size, genotypes were binarized based on the presence/absence of *APOEε*4. Age and educational level were normally distributed and they were summarized as mean ± standard deviation. The student’s t-test was used to compare these data between the carriers and non-carriers of *APOEε4* allele. MMSE, Aβ_1-42_ oligomer and anti-Aβ antibody levels were not normally distributed, and they were summarized as median and interquartile range (IQR: 25_th_-75_th_ percentile). The Mann–Whitney test was used to compare MMSE, Aβ_1-42_ oligomer and anti-Aβ antibody levels between the carriers and non-carriers of *APOEε*4 allele. The statistical power of the study was estimated for comparing the two independent groups using a two-sided t-test. Fisher’s exact test was used to compare gender between the carriers and non-carriers of *APOEε*4 allele. A general linear regression model was applied to correlate anti-Aβ antibody levels and *APOEε*4 carrier status, adjusting for age, sex, serum amyloid beta levels, and the MMSE scores. Spearman’s correlation coefficient was used determine the association between MMSE scores and anti-Aβ antibody levels. For all analyses, *p* < 0.05 was considered statistically significant.

## Results

### *APOE* genotypes and the level of anti-aβ antibodies

The MMSE score of the 93 AD patients enrolled in the study (35 males and 58 females, age: 76.71 ± 6.30 years) was 18.99 ± 3.79 (educational level: 8.16 ± 3.36 years). The median value of Aβ1-42 oligomer was 0.01 (0.01–1.07) pg/mL.

**Figure 1:**
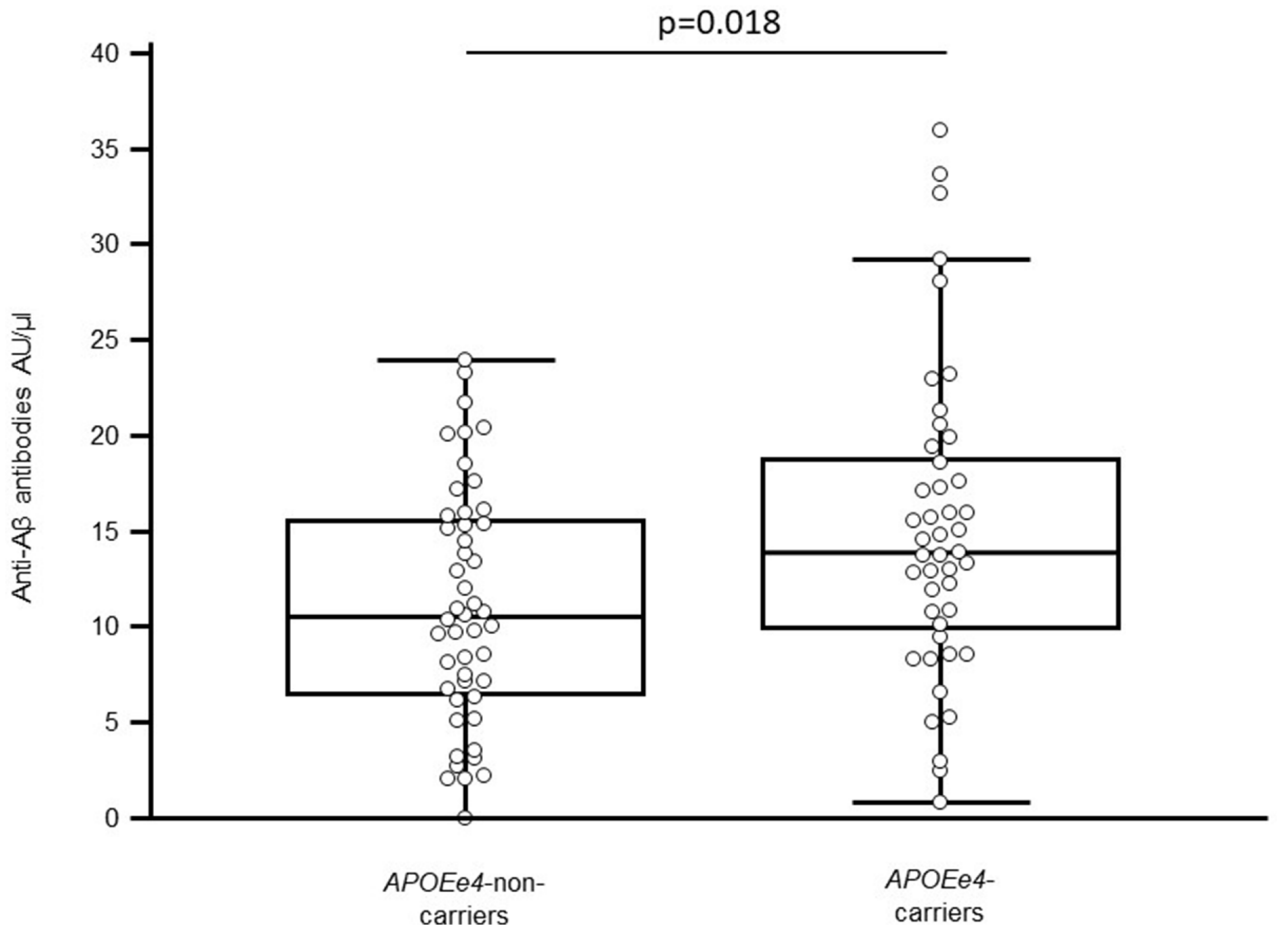
*APOEε4*-non-carriers among AD patients.

Among the 93 AD subjects enrolled in the study, there were 45 (48%) *APOEε*4-carriers, defined by the presence of at least one ε4 allele—either in the heterozygous state (ε2/ε4: 1; ε3/ε4: 38) or homozygous state (ε4/ε4: 6). The remaining 48 subjects (52%) were non-carriers (ε2/ε2: 1; ε2/ε3: 4; ε3/ε3: 43). These results are summarized in Table 1. As shown in Figure 1, *APOEe*4-carriers had significantly higher ELISA reactivity to Aβ_1-42_ oligomer (14.25 AU/μL; 9.82–19.00) than non-carriers (10.52 AU/μL; 6.52–15.57; Mann–Whitney test, *p* = 0.018), with a statistical power of 78%. After adjusting for age, sex, Aβ levels in serum, and the MMSE scores, regression analyses showed marginally significant association between *APOEε*4 carrier status and levels of anti-Aβ antibodies (*p* = 0.050, R^2^ = 0.15). These results should be interpreted cautiously.

### MMSE scores, anti-aβ levels, and *APOE* genotypes

MMSE scores were not associated with the levels of naturally occurring anti-Aβ antibodies (Spearman’s correlation test, *p* = 0.127; 95% confidence interval: −0.132–0.781; correlation coefficient: 0.428), or with the *APOEε*4 carrier status (Mann–Whitney test, *p* = 0.846; *APOEε4*-carriers: median MMSE score: 19; *APOEε4*-non-carriers: median MMSE score: 20). These results are summarized in Table 1. No correlation was found between anti-Aβ antibodies and Aβ_1-42_ oligomer (Spearman’s correlation test, *p* = 0.100; 95% confidence interval: −0.0347–0.374; correlation coefficient: 0.177).

## Discussion

The results presented here show a distinct association between the presence of *APOEε*4 allele and higher levels of naturally occurring anti-Aβ antibodies. There are at least two possible explanations for the observed associations. The *APOEε*4 allele could itself somehow affect anti-Aβ antibody responsiveness. Alternatively, linkage disequilibrium between the *APOEε*4 allele and an allele of another as-yet-unidentified immune response gene on chromosome 19 may give rise to the associations observed.

To our knowledge, no studies have reported the influence of *APOE* genotypes on the level of naturally occurring anti-Aβ antibodies. However, *APOEε*4 allele has been shown to be associated with increased *APOE* expression (Griswold et al., 2021), which is positively associated with anti-Aβ antibody titer in patients immunized with Aβ_1-42_ (van Olst et al., 2025). Thus_,_ it follows that the *APOEε*4 allele could influence the level of naturally occurring anti-Aβ antibodies observed in this investigation—assuming immunological mechanisms underpinning the generation of naturally occurring anti-Aβ antibodies and those generated after immunization with Aβ are similar.

At first glance, the results reported here appear to be counterintuitive. Since *APOEe*4 is the strongest known genetic risk factor for AD, and naturally occurring anti-Aβ antibodies reduce Aβ plaque burden (Kellner et al., 2009), one would expect *APOEε*4 carriers to be associated with lower levels of antibodies compared to non-carriers. On the other hand, *APOEε*4-spurred aggregation and deposition of Aβ (Jackson et al., 2024) would increase the Aβ antigenic load, leading to the generation of higher levels of anti-Aβ antibodies.

This study has a few limitations. The cross-sectional design used in the study measures association between *APOE* genotypes and anti-Aβ antibodies, but does not prove causation. Since we did not have PET or CSF data, we cannot rule out that higher antibody levels reflect higher Aβ antigenic load in the brain. The anti-Aβ IgG levels were not normalized to total IgG levels. Thus, higher anti-Aβ IgG levels in the *APOEε4* group could reflect the possible association of the ε4 allele with higher total IgG levels.

This is the first report documenting the involvement of *APOE* genotypes in humoral immunity to Aβ. Owing to significant racial/ethnic differences in the magnitude of *APOEε*4-related risk of AD (Jackson et al., 2024), a study involving a large multiethnic study population is needed to conclusively determine the role of the *APOEε*4 determinant in the generation of naturally occurring anti-Aβ antibodies.

## Data Availability

The raw data supporting the conclusions of this article will be made available by the authors, without undue reservation.
